# The quantitative and qualitative histomorphological structure of human stapes footplate

**DOI:** 10.1038/s41598-026-43700-8

**Published:** 2026-03-19

**Authors:** Max Kemper, Erdem Türkeli, Anne Kluge, Susanne Isabella Günther, Thomas Zahnert, Marcus Neudert

**Affiliations:** Department of Otolaryngology, Head and Neck Surgery, Technische Universität Dresden, Fetscherstrasse 74, 01307 Dresden, Germany

**Keywords:** Histomorphology, Human stapes footplate, Bone and cartilage layer, Anatomy, Medical research, Bone, Bone development, Cartilage development

## Abstract

This study analyzes the histomorphology of human stapes footplates, focusing on the footplate (FP), to enhance reconstructive otosurgery. Seven human stapes specimens obtained during pathological dissections were histologically processed. Tissue layer thicknesses (bone and cartilage) were measured at five points in longitudinal sections and three points in cross sections. Additional analyses of surrounding tissue area up to 100 μm from each point were conducted. A total of 1400 individual measurements were included in the analysis of the human FP, comprising 350 measurements each for cartilage and bone layer thickness, as well as the determined cartilage and bone areas. The FP consisted of a flat epithelial layer, a bone layer facing the tympanic cavity, and a cartilage layer. The central total thickness was 118.06 μm, increasing by 36% toward the periphery. Bone composition was 40% centrally and larger peripherally at 56%. Cross sections showed more homogeneous distribution, with a total thickness of 73.44 μm and 42% bone. This first detailed qualitative and quantitative topographical tissue analysis of the FP confirms a bilayered bone-cartilage structure with increasing bone thickness toward the periphery. The findings provide critical insights into prosthetic coupling sites and support the development of optimized reconstructive strategies in middle ear surgery.

**Supplementary Information:**

The online version contains supplementary material available at 10.1038/s41598-026-43700-8.

## Introduction

**Fig. 1:**
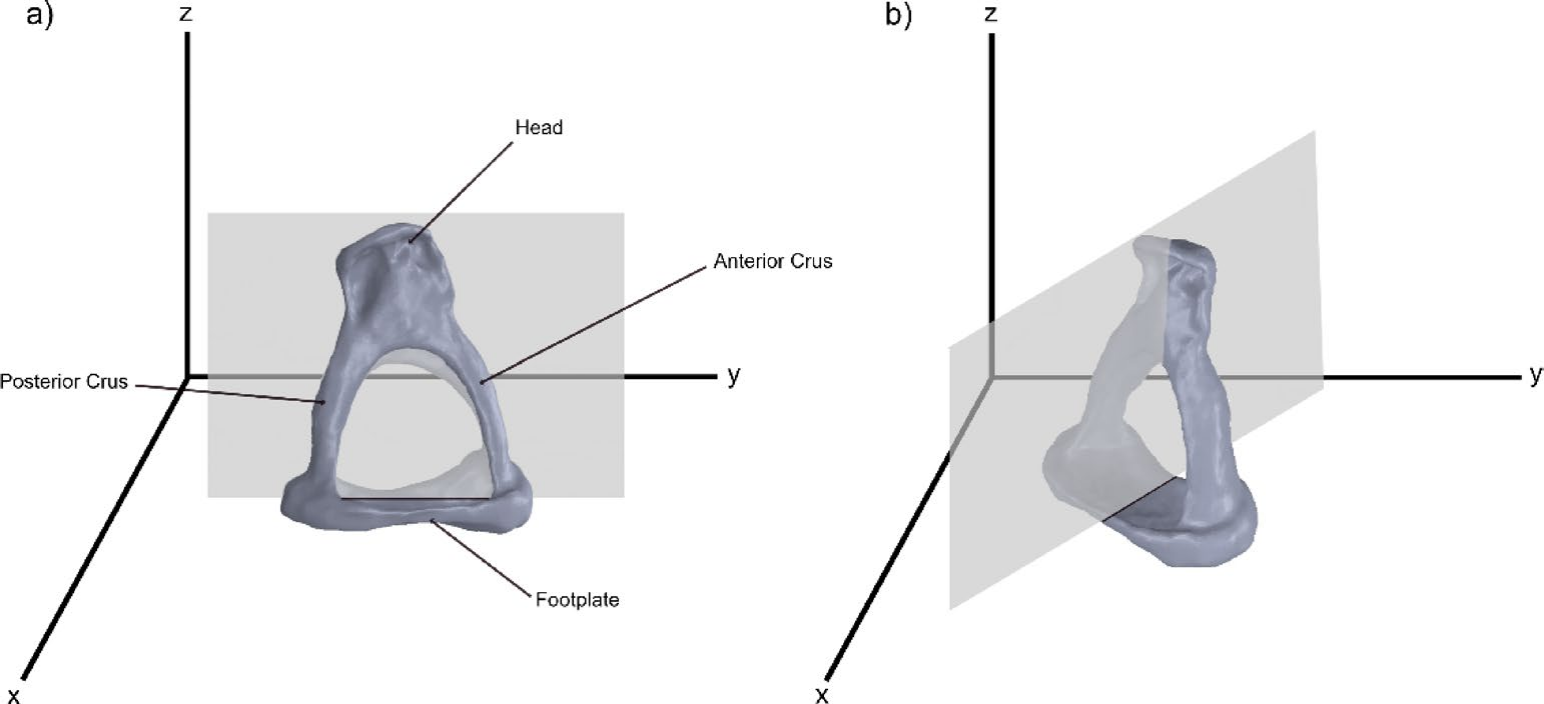
Schematic representation of the cutting planes through the human stapes in the longitudinal direction (**a**) and in the cross direction (**b**).

The fundamental prerequisite for optimal auditory perception is the morphological and functional integrity of the sound conduction system, which principally comprises the tympanic membrane, the malleus, the incus and the stapes, subdivided into the stapes suprastructure and the stapes footplate (FP) (Fig. 1a). The capacity of this ossicular chain to vibrate, a process further influenced by the articulated connections, the eardrum, and the annular ligament, and the associated muscles, determines the quality of sound transmission. Even small changes to these structures can diminish sound transmission; reconstructive procedures have a similar effect, though the magnitude varies. A substantial corpus of research conducted on temporal bone specimens featuring an intact ossicular chain has demonstrated that the FP, in particular, possesses the capacity to move in all directions, contingent on the frequency^1–5^. Such a complex movement pattern imposes particular demands on the annular ligament and, in the context of ossicular chain reconstruction, on the stability of the connection between the FP and the prosthesis^5^.

Furthermore, it has been known since at least 2010 that growth factors can induce osteoinduction and subsequent osseointegration of titanium prostheses on the FP in mammals^6,7^. This process necessitates active bone metabolism in the middle ear, including the FP. The configuration of the stapes is crucial for selecting the most suitable prosthesis, particularly in the context of the total ossicular replacement prosthesis (TORP) or the bimodular prosthesis concept^6,8^. The optimal prosthesis positioning on the FP is essential to ensure optimal balance between the prosthesis’ load and the forces that facilitate sound transmission^9–11^. Consequently, a comprehensive understanding of the histomorphological structure of the FP is imperative for the purpose of achieving optimal hearing rehabilitation.

While previous research has addressed the macroscopic morphology of the stapes, no prior studies have offered detailed qualitative and quantitative insights into the structural composition of the FP^12^. This study presents the precise quantitative and qualitative histomorphological structure of the human FP for the first time.

## Materials and methods

Seven human stapes were explanted; five were prepared for histological analysis in longitudinal sections and two in cross sections (Fig. 1).

### Sample collection and preparation of histological sections

All methods were carried out in accordance with relevant guidelines and regulations, including the Declaration of Helsinki and applicable German legislation governing the use of human tissue for research. The study protocol, including the use of human temporal bone specimens and subsequent extraction of the stapes, was approved by the Ethics Committee of the Technische Universität Dresden (Ethikkommission an der TU Dresden; approval no. EK 59022014). Seven human stapes footplates were obtained from anonymised adult human temporal bone specimens that had previously undergone middle-ear sound transmission measurements within this approved protocol. At the end of these measurements, and following fixation of the temporal bones in a 4% formaldehyde solution for one day, an exploratory endaural tympanotomy was performed and the entire stapes was carefully removed while keeping the footplate intact. Only those ossicles were considered healthy in which microscopic examination of the ear canal, eardrum and tympanic cavity during the experimental procedures had revealed no pathological findings.

Each specimen was then subjected to the following histological processing steps: fixation, decalcification with an EDTA-based chelating agent for one week and embedding in hot paraffin. The subsequently cooled blocks were processed into 1–2 μm thick histological sections using a rotary microtome (RM 2125RT, Leica, Wetzlar, Germany). Two section planes were defined to determine the width and length of the FP. For this purpose, longitudinal or transverse sections were prepared from different stapes samples. The aim was to obtain a sequence of 20 longitudinal sections with both stapes crura (SC) visible and 50 transverse sections (Fig. 1a,b). Subsequently, automated haematoxylin-eosin (HE) staining was performed in the staining machine (Tissue Tek Prisma/Tissue Tek Film, Sakura, Alphen aan den Rijn, The Netherlands).

### Computer-aided measurement of the histological samples

**Fig. 2:**
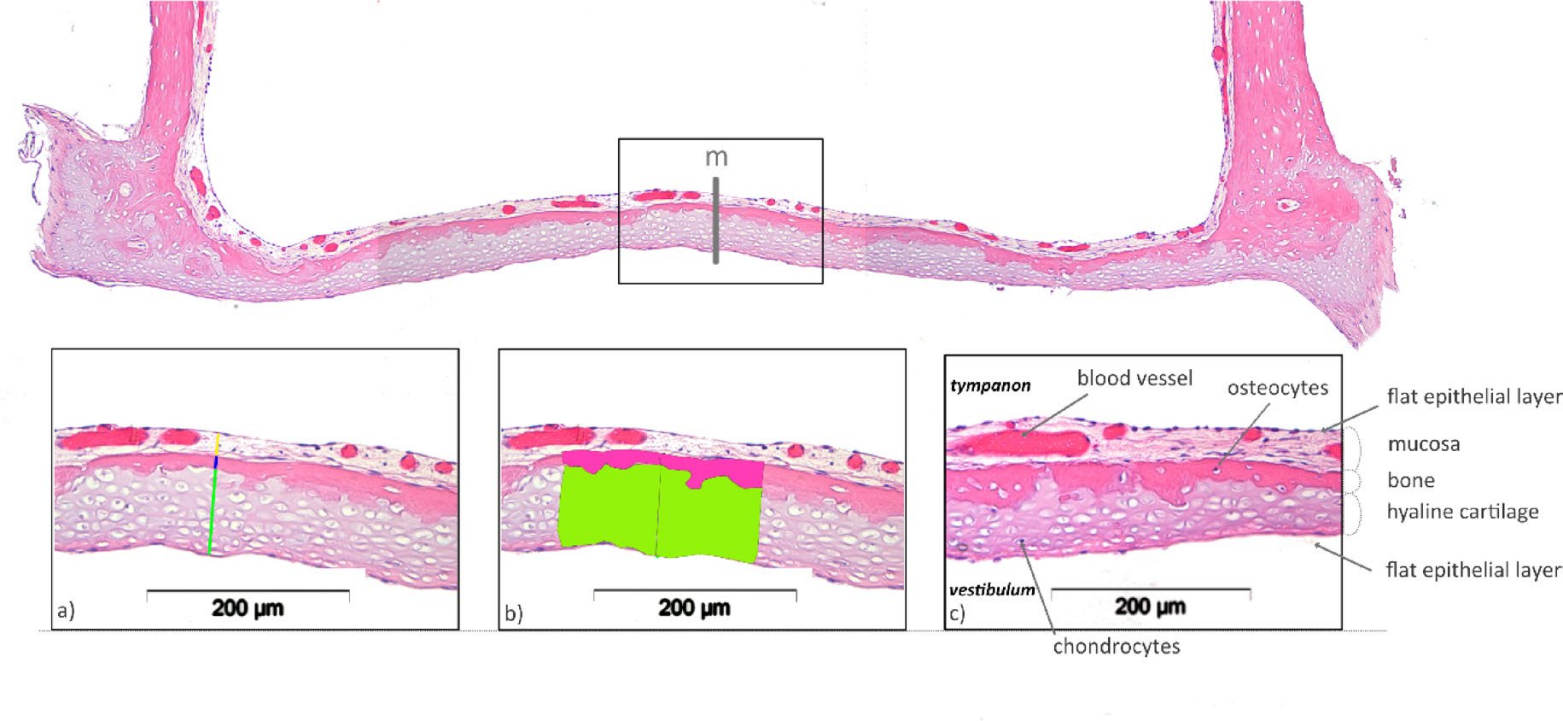
Illustration of computer-assisted thickness and area determination in the histological section preparation at measuring point m in longitudinal section. (**a**) Measurement of layer thicknesss (blue line = bone layer; green line = cartilage layer); (**b**) Measurement of bone area (pink) and cartilage area (green) (**c**) histomorphological description.

To accurately measure the entire FP, measurements were taken both at and around defined points from defined areas (Fig. 2). The individual sections were first examined microscopically for tissue integrity. Histological sections with process-related artefacts such as wrinkling, tissue loss and section overlap were excluded. All histological sections were measured with a reflected-light microscope (BX61, Olympus, Hamburg, Germany) and the corresponding computer-assisted analysis program (cellF, Olympus, Hamburg, Germany).

Using a digital camera (Color View II, Olympus, Hamburg, Germany) integrated into the microscope, all samples were aligned approximately with the long axis of the stapes footplate of the FP at the lowest magnification, photographed and evaluated using a computer-assisted analysis program. The overview images of the FP were taken at 2 ×, 4 × and 10 × magnification. The abovementioned analysis software automatically adjusts the measurements and measuring points, as well as the corresponding lengths and areas for each magnification to avoid shifting the position of the measuring.

Initially, the total width of the stapes was defined as the horizontal width of the FP and determined at 4 × magnification of the specimen. The measurements of the layer thickness (in µm) at the defined points and in the defined areas (in µm^2^) were carried out at 20 × magnification. The distances for the layer thickness at the defined measuring points were drawn in the recorded images and the defined layer areas were marked (Fig. 2).

### Measurement and analysis of the FP in longitudinal and cross-sectional views

**Fig. 3:**
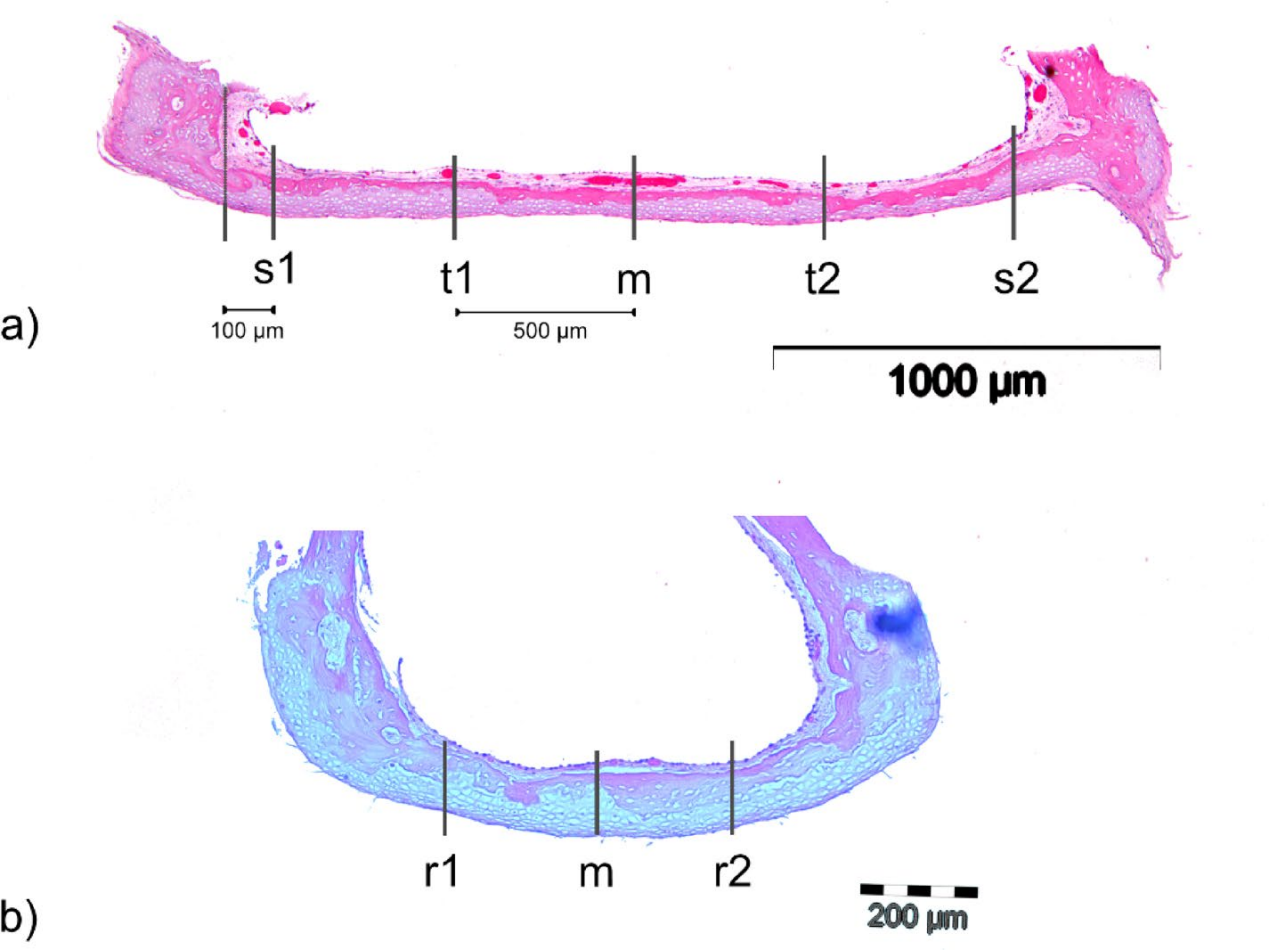
Schematic representation of the measuring points in the stapes footplate on the histological section in the longitudinal (**a**) and cross-sectional (**b**) directions.

The FP was analysed in both longitudinal and cross-sectional views. As illustrated in Fig. 3, measurements in the longitudinal section were taken at five specific points: s1, t1, m, t2, and s2. The central measurement point, m, corresponds to the midpoint calculated from the total length of the FP. Points t1 and t2 were located 500 μm to the right and left of m, respectively, while s1 and s2 were defined as points positioned 100 μm from the base of the SC to the center of the FP. To ensure accurate measurements, a horizontal auxiliary line was drawn beneath the base plate, followed by vertical lines positioned at each measurement point, intersecting the footplate perpendicularly to the auxiliary line.

In the cross section, as shown in Fig. 3b, three measurement points were defined: r1, m, and r2. The points r1 (left) and r2 (right) were positioned midway between the center of the footplate and the inner edge of the footplate wall, while m represented the calculated central point based on the total width of the footplate.

#### Determination of tissue thickness

At each measurement point, the thicknesses of the cartilage layer (CLT) and bone layer (BLT) were determined along the vertical measurement lines, with their sum resulting in the total tissue layer thickness (TLT).

#### Determination of tissue areas

Since the bone-cartilage interface along the footplate meanders, the tissue thicknesses at directly adjacent points sometimes show large differences. To eliminate errors resulting from this, additional tissue areas were defined and evaluated around the measuring points.

The surrounding bone and cartilage areas were assessed by measuring a 200 μm-wide region, extending 100 μm to the left and right of each measurement point (Fig. 2). At each of these locations, namely s1, t1, m, t2, and s2 in the longitudinal sections, as well as r1, m, and r2 in the cross sections, the cartilage area (CA) and bone area (BA) were identified (Fig. 3). The height of the measured region varied depending on the specific tissue structures, as depicted in Fig. 2.

### Statistical analysis

Statistical analysis was conducted using Excel (Version 2024, Microsoft, USA) and SPSS (Version 30, IBM, USA). A two-sided significance level of 5% (α = 0.05) was used for all hypothesis tests. Because the anterior/posterior orientation was not preserved in longitudinal sections, s1 and s2 were analysed as unlabeled edge positions. Likewise, in cross sections, superior/inferior orientation was not preserved and were treated as unlabeled, mirrored edge positions. Because only two independent footplates were available for cross-sectional analyses, we refrained from formal equivalence testing in cross sections to avoid underpowered inferences. Descriptive statistics, including arithmetic mean, median, standard deviation, and minimum and maximum layer thickness, were calculated. To analyze the relationships between cartilage thickness, bone thickness, and area at various measurement points, Pearson correlations were computed. Because totals equal the sum of their components (TLT = BLT + CLT; TA = BA + CA), correlations between totals and single components are arithmetically coupled and were not used for mechanistic inference. We therefore emphasised component–component associations (BLT–CLT; BA–CA) and symmetry across mirrored positions. Differences in mean layer thickness across positions were assessed using one-way repeated-measures ANOVA. Where necessary, Bonferroni-corrected post-hoc tests were applied to control for type I error in multiple comparisons. To evaluate whether the two edge positions can be considered practically equivalent, we performed equivalence testing using the two one-sided tests (TOST) procedure on paired differences (s1–s2) for each outcome (TLTl, BLTl, CLTl). The equivalence margin was pre-specified as Δ = 50 μm, reflecting the average variability observed at the edge positions across specimens. Equivalence was concluded if the 90% confidence interval of the mean paired difference lay entirely within [− 50 μm, + 50 μm] (α = 0.05).

## Results

A total of seven FPs were analysed, five in longitudinal and two in transverse direction. Across the five longitudinal footplates included for analysis, 76 sections were prepared; 60 (78.9%) were analysed and 16 (21.1%) were discarded due to histological artifacts or position mismatch. One additional footplate (FP5) yielded only central sections and was excluded at the specimen level. In cross sections, two footplates yielded 44 sections in total (FP1: 12; FP2: 32). All sections were evaluable and included (0% excluded).

**Fig. 4:**
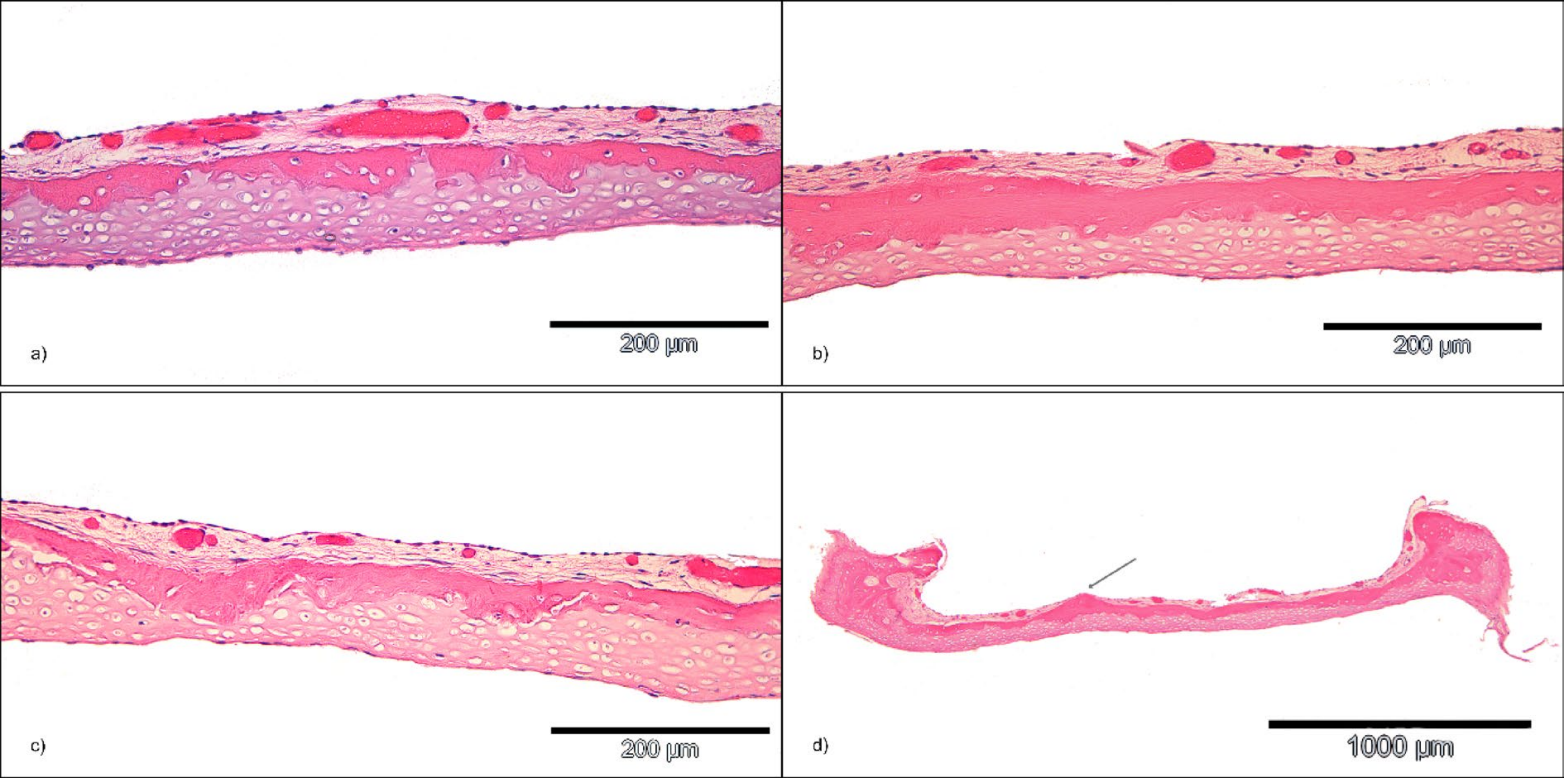
Presentation of the various histomorphological characteristics and special features of the stapes footplate (HE staining), the bone layer and cartilage layer at the measuring point m (**a**–**c**), (**a**) small bone layer thickness, large cartilage layer thickness, (**b**) almost equally thick bone and cartilage layer, (**c**) markedly varying layer thicknesses of bone and cartilage with relatively constant overall thickness (**d**) Protruding crista intercruralis in the centre of the FP, cross section.

During histological analysis in both longitudinal and cross sections, significant variations in the proportion of bone and cartilage within the FP were observed (Fig. 4a–c). The values presented are reported as mean value ± standard deviation (MV ± SD). For each histological section of a single FP, layer thicknesses and areas were measured, and an average was calculated. These averages were then aggregated across all sections to determine a single mean value for each stapes preparation. Additionally, the crista intercruralis was identifiable in the histological sections of one FP specimen (Fig. 4d).

### Tissue layer thicknesses and areas in longitudinal sections

The number of measured values of BLTl and CLTl, as well as the corresponding areas varied depending on the number of evaluable histological sections per stapes preparation. The total number of bone and cartilage layer thicknesses and areas determined was 350. This resulted in a total of 1400 individual measurements being analysed. The MV, SD and the lowest and highest thickness or area were determined from the measured values of each measuring point.

#### Tissue layer thickness

**Fig. 5:**
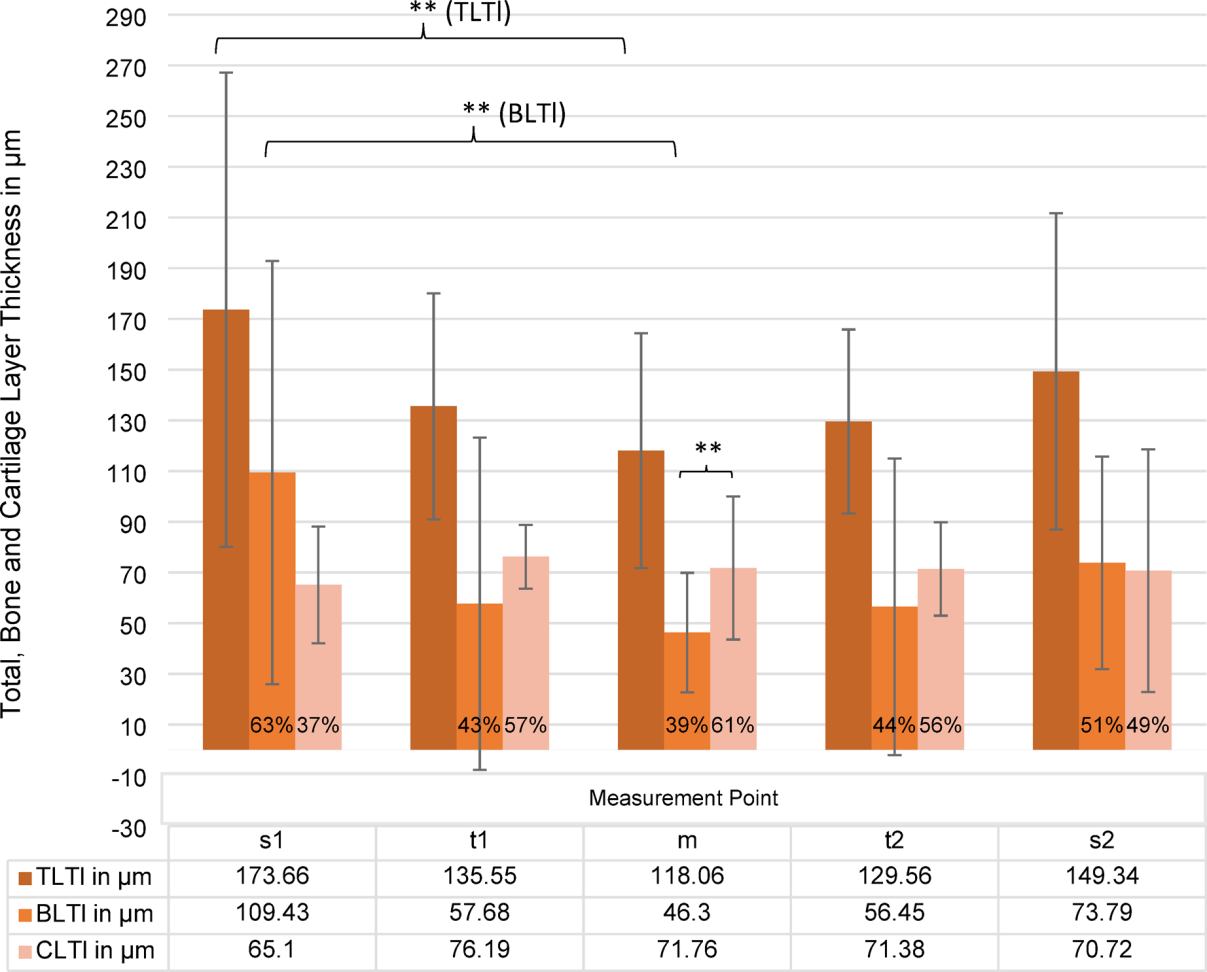
Thickness distribution (in µm) of total layer thickness (TLTl), bone layer thickness BLTl (brown) and cartialge layer thickness CLTl (light orange) in longitudinal section across different measurement points (s1, t1, m, t2, s2). The percentage values indicate the proportion of each component within the total thickness. Error bars represent standard deviations. A statistically significant difference (**p < .01) is observed between groups s1 and m. Additionally, a significant difference was found between CLTl and BLTl at measurement point m (**p < .01), while no significant differences were detected for other comparisons.

The data for the analysis of the layer thicknesses is presented in Fig. 5. At measuring point m, the mean total layer thickness (TLTl) was 118.06 μm ± 46.35. In the ANOVA performed in order to make a comparison between the different layer thicknesses, a significant difference in the TLTl was identified between measuring point m and measuring point s1 (*p*<.001). For BLTl, a significant difference was identified between measuring point m and the more peripheral measuring point s1 (*p* < .001), but not to measuring point s2 (*p *=.306). Additionally, no statistically significant variation was observed in BLTl at t1 and t2 measurement points. For CLTl, no statistically significant differences were identified between the central and peripheral measurement points s1/2 and t1/2.

A comparison across FPs revealed significant disparities in TLTl between FP4 and FP1 (*p* < .001) and FP2 (*p* = .034). BLTl also differed between FP4 and FP1 (*p* = .002) and CLTl at FP2 differed from FP4 (*p* = .029).

Using the pre-specified equivalence margin (Δ = 50 μm), s1 and s2 were equivalent for TLTl (mean difference s1–s2 = − 13.7 μm; 90% CI − 47.2 to 19.8) and CLTl (− 11.3 μm; 90% CI − 32.5 to 9.9), but not equivalent for BLTl (+ 37.6 μm; 90% CI − 11.4 to 86.5). Thus, within a histologically meaningful tolerance, total and cartilage thickness did not differ between the two edges, whereas bone thickness showed greater variability across footplates. These findings complement the ANOVA results, where some within-footplate contrasts (e.g., BLTl in FP4; CLTl in FP1) reached significance without indicating a systematic edge effect across specimens.

At the center (m), mean BLTl was 46.30 ± 23.56 μm, lower than CLTl (71.76 ± 28.20 μm; *p* = .001), corresponding to 39% BLTl and 61% CLTl of TLTl. Both layers tended to increase toward the SC: TLTl at t1 and t2 was + 14% and + 9% relative to the center, respectively, without statistical significance (*p* = .100). The proportion of BLTl was larger at t1 (48%) and at t2 (47%). Near the SC, the BLTl proportion was 46% at s1 and 26% at s2, with absolute BLTl means of 109.43 ± 83.45 μm at s1 and 73.79 ± 41.96 μm at s2 (overall *p* = .506). At the single-footplate level, BLTl at s1 exceeded s2 in FP4 (*p* = .007). For cartilage, an s1–s2 difference was observed only in FP1 (*p* < .001); the remaining footplates showed no substantial s1–s2 variation.

#### Tissue areas

**Fig. 6:**
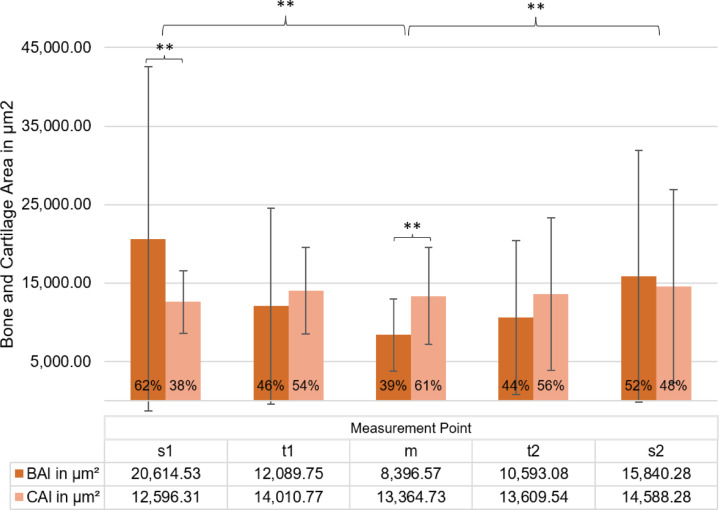
Bone area BAl (brown) and cartilage area CAl (light orange) in longitudinal section (µm^2^) across measurement points (s1, t1, m, t2, s2). Percentage values indicate each component’s proportion within the total area (TAl). Error bars represent standard deviations. Statistically significant differences in TAl (**p < .01) were observed between measurement points s1 and m, as well as s2 and m. Additionally, BAl and CAl differed significantly at measurement points m and s1 (**p < .01), while no other comparisons showed significant differences.

The analysis of total area (TAl), bone area (BAl), and cartilage area (CAl) revealed structural variations across different footplate regions, which are shown in Fig. 6. In the central region, TAl was lowest, with BAl contributing 38% and CAl 62%. ANOVA showed significant differences in TAl between FP4 and FP1, FP2, and FP6 (*p* < .010), in BAl between FP4 and FP1 (*p* = .001) and FP6 (*p* = .010), and in CAl between FP4 and FP2 (*p* = .005). At t1 and t2, Tal, BAl and Cal were larger, but CAl differed significantly between t1 and t2 in FP1 (*p* = .004) and FP3 (*p* = .042). At s1 and s2, TAl peaked, with significant differences in TA for FP1 (*p* = .003) and FP6 (*p* = .044), in BA for FP4 (*p* = .003), and in CAl for FP1 (*p* < .001).

BAl was larger at s1 and s2, corresponding to differences of 154% and 86% (*p* < .001, *p* = .008, while CAl showed only small differences of -11% at s1 and 13% at s2 (*p* = 1.000, *p* = .590). The proportion of BAl was 53% at t1 and 43% at t2, compared with 64% at s1 and 51% at s2, indicating a shift in structural composition.

#### Correlation analysis of tissue thickness and area measurements

To evaluate symmetry between longitudinal positions, we correlated measurements between t1/t2 and s1/s2. BLTl showed moderate correlations across both pairs (t1 vs. t2: *r* = .508, *p* < .001; s1 vs. s2: *r* = .544, *p* < .001). TLTl also correlated across mirrored positions (t1 vs. t2: *r* = .564; s1 vs. s2: *r* = .651; both significant), whereas CLTl did not exhibit significant symmetry (both pairs n.s.). We focused on component–component relationships within positions and across footplate subgroups (FP1, FP2, FP3, FP4, FP6). At the peripheral edge s1, total thickness closely tracked bone (BLTl–TLTl *r* = .979, *p* < .001), while subgroup analyses revealed significant negative BLTl–CLTl trade-offs in FP1 and FP3, and a cartilage-driven contribution to TLTl in FP6 (CLTl–TLTl, *p* < .001). At t1, both components contributed to the total (BLTl–TLTl, *p* < .001; CLTl–TLTl, *p* < .001, overall), with FP1 again showing a negative BLTl–CLTl association and FP6 a positive CLTl–TLTl link. Centrally (m), bone and cartilage co-thickened (BLTl–CLTl *r* = .310, *p* < .001), and both correlated strongly with TLTl (BLTl–TLTl, *p* < .001; CLTl–TLTl, *p* < .001). At t2, several subgroups (FP1–FP3) showed negative BLTl–CLTl correlations, consistent with local trade-off, while CLTl–TLTl was positive in FP4/FP6.

At the contralateral edge s2, patterns mirrored s1: strong BLTl–TLTl overall, with negative BLTl–CLTl in FP3 and positive CLTl–TLTl in FP1/FP4/FP6.

Across positions and footplate subgroups, total area (TAl) closely tracked bone area (BAl). BAl–TAl correlations were consistently high (frequently *r* ≥ .95; several instances *r* = .98–0.999; all significant where tested), indicating that TAl is largely bone-driven. By contrast, cartilage area (CAl) showed more variable coupling with TAl: CAl–TAl ranged from moderate (*r* = .58–0.71) to very strong (*r* = .89–0.97), depending on subgroup/position. In multiple groups, BAl and CAl were inversely related (BAl–CAl negative correlations up to *r* = − .797; all significant where indicated), consistent with a local trade-off between bone and cartilage contributions to the same footprint.

Mirrored-position symmetry analyses revealed moderate bilateral symmetry for BAl and TAl but weaker or absent symmetry for CAl. Specifically, BAl correlated between s1–s2 (*r* = .651, *p* < .001), and t1–t2 (*r* = .574, *p* < .001); TAl likewise showed s1–s2 (*r* = .682, *p* < .001) and t1–t2 (*r* = .555, *p* < .001). In contrast, CAl symmetry was only weakly expressed at s1–s2 (*r* = .435, *p* < .001), and non-significant at t1–t2. Together, these patterns indicate that area symmetry is predominantly set by bone, with cartilage exhibiting greater local variability.

### Tissue layer thicknesses and areas in cross sections

Cross sections were available from two stapes footplates (FP1, FP2). Across both footplates, 44 evaluable histological sections per measurement point (r1, m, r2) were obtained. As superior/inferior orientation was not preserved, r1 and r2 were treated as unlabeled, mirrored edge positions. The number of measured BLT and CLT values varied depending on the number of evaluable histological sections per stapes preparation. In total, 132 bone and cartilage layer thicknesses were determined, alongside 132 respective area measurements, resulting in a dataset of 528 values for analysis.

#### Tissue layer thickness in cross section

**Fig. 7:**
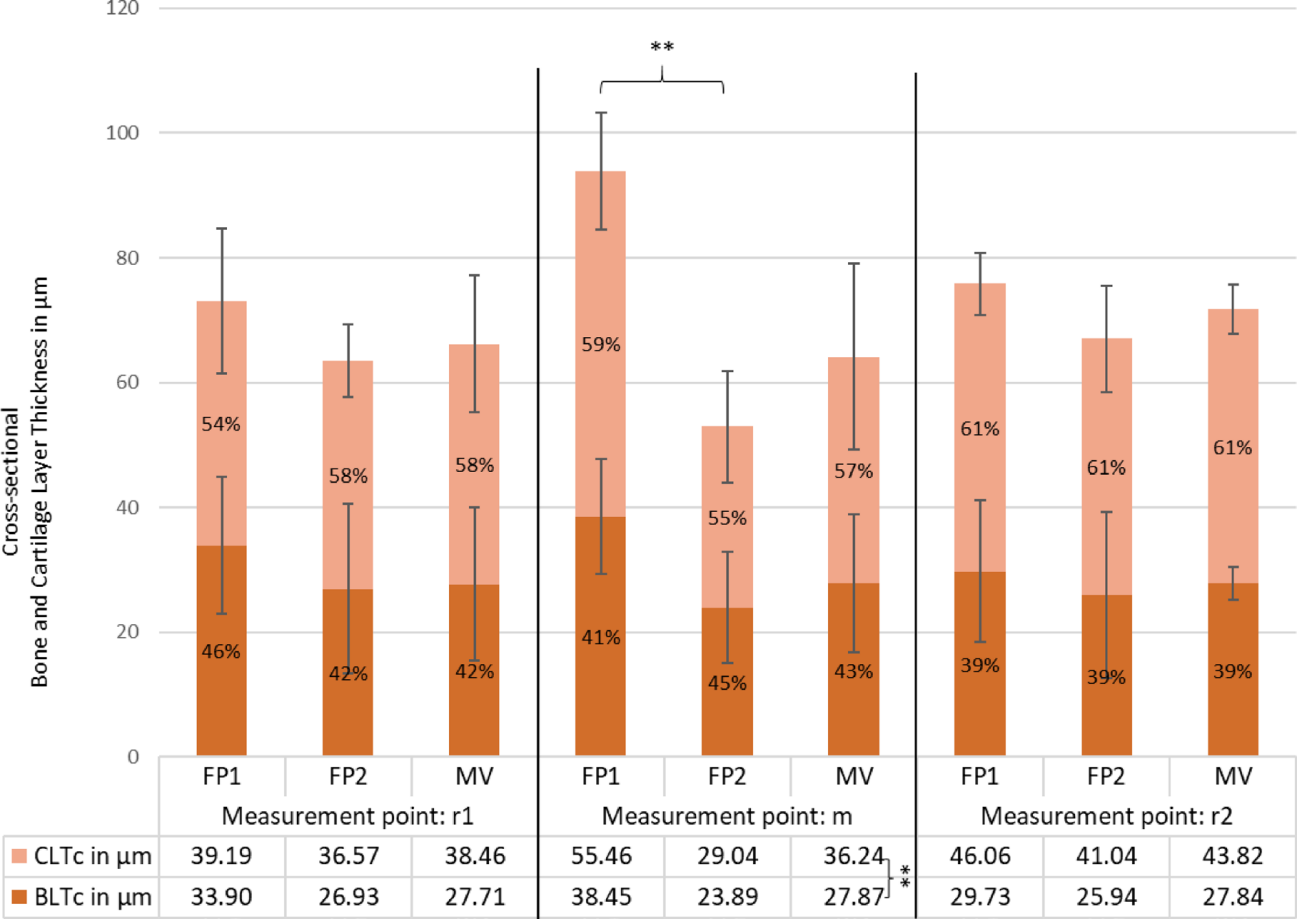
Cross-sectional tissue thickness (µm) of the stapes footplates (FP1 and FP2) and their mean value (MV) at three different measurement points (r1, m and r2). CLTc (cartilage layer thickness) is shown in light orange, and BLTc (bone layer thickness) in brown. Percentage values indicate the proportion of each component within the total thickness. Error bars represent standard deviations. A statistically significant difference (**p < .01) was observed between FP1 and FP2 at the midpoint m (98.91 μm vs. 52.93 μm). CLTc and BLTc also differed significantly in the MV (*p* < .001).

The mean total layer thickness in cross-section (TLTc) at the center of the footplate (m) was 64.12 ± 22.19 μm; BLTc and CLTc accounted for 43% and 57%, respectively (Fig. 7). At the centre, FP1 was significantly thicker than FP2 (93.91 μm vs. 52.93 μm; *p* < .001). Across positions, FP1 showed a central maximum and became smaller toward the edges (r1 = 73.09 μm, r2 = 75.79 μm), whereas FP2 showed the opposite pattern, being thinner in the centre and thicker toward the edges (r1 = 63.50 μm, r2 = 66.98 μm). When averaging across both footplates, mean TLTc was slightly larger toward the edges (m = 64.11 μm, r1 = 66.17 μm, r2 = 71.66 μm), but these differences were not statistically significant (*p* = .403). Given the small number of independent specimens (*n* = 2), no formal equivalence testing was performed for cross sections; instead, we report descriptive patterns and correlations as indicators of symmetry (see Sect. “Correlations of tissue layer thicknesses and areas”).

#### Tissue areas in cross section

**Fig. 8:**
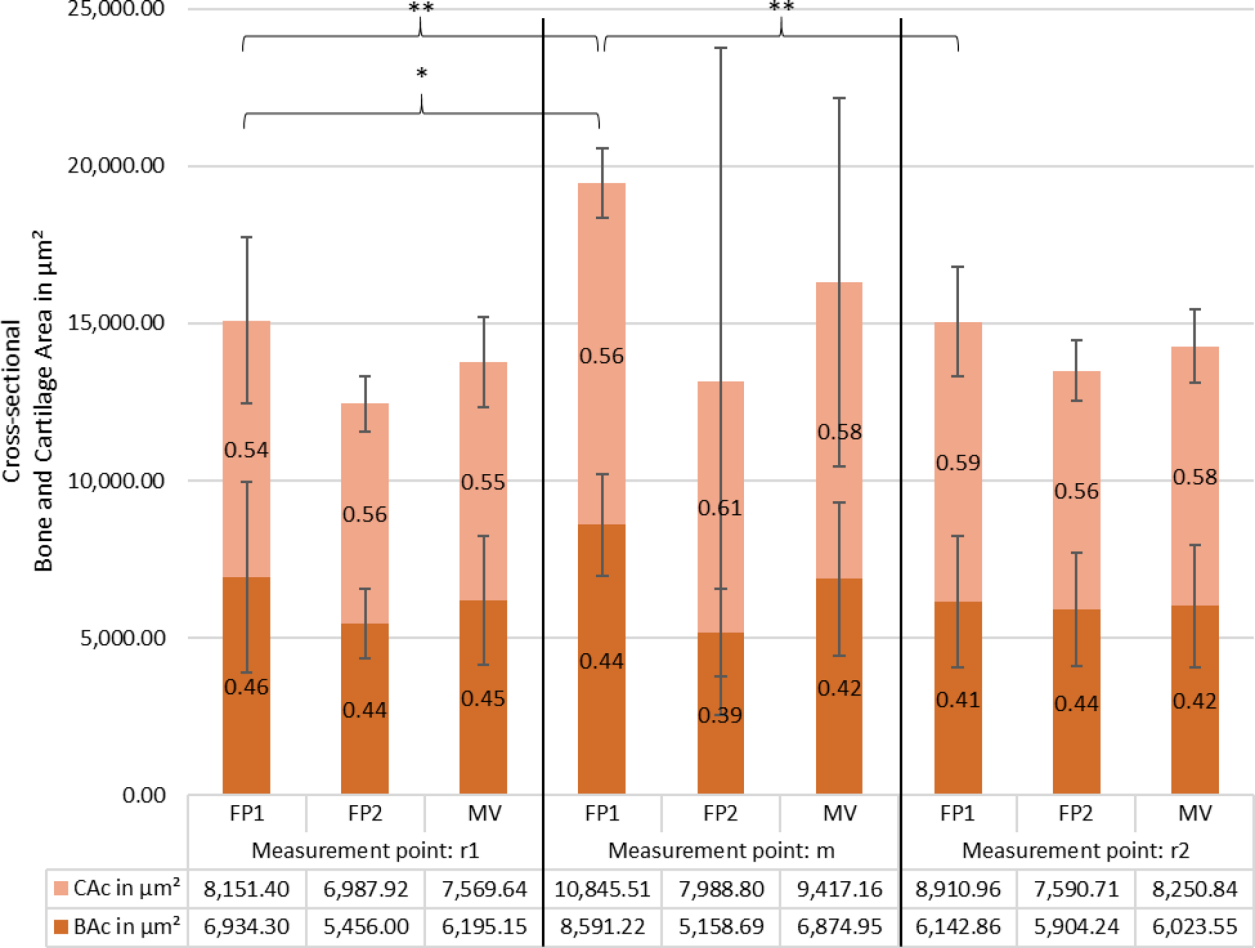
Tissue area distribution (in µm^2^) of bone area (BAc, brown) and cartilage area (CAc, light orange) in stapes footplates FP1 and FP2 at r1, m, r2, with mean values (MV) shown. Percentage labels indicate the proportion of each component within the total area (TAc). Error bars represent standard deviations. In FP1, TAc was significantly lower at r1 and r2 compared with m (*p* < .001). CAc was smaller at r1 (*p* = .008) and tended to be smaller at r2 (*p* = .076); BAc did not change significantly across positions. In FP2, no significant differences in TAc, BAc, or CAc were observed across positions.

This indicates that the total tissue area was significantly larger at the center compared to the lateral measurement points (Fig. 8). While bone area in cross section (BAc) did not significantly differ between measurement points, cartilage area in cross section (CAc) at FP1 exhibited a significant decrease at r1 (22694.16 μm^2^, *p* = .008) and a trend towards a decrease at r2 (− 1934.55 μm^2^, *p* = .076), suggesting a relative thinning of cartilage in the lateral regions. In contrast, FP2 showed no significant differences in TAc, BAc or CAc across measurement points, indicating a more uniform tissue distribution. These findings suggest specimen-dependent patterns, with FP1 exhibiting central enrichment, particularly of cartilage, whereas FP2 maintains a more even distribution.

#### Correlations of tissue layer thicknesses and areas

To evaluate symmetry between tissue distribution and mirrored edge positions, we correlated measurements at r1 and r2. TLTc and CLTc were significantly correlated across r1 and r2 (*p* = .040), and TAc and CAc showed analogous symmetry (*p* = .034), indicating comparable tissue distribution at both edges despite the lack of superior/inferior labelling.

Because totals equal the sum of their components (TLTc = BLTc + CLTc; TAc = BAc + CAc), correlations between totals and components are mathematically coupled and not mechanistically informative. We therefore focused on component–component relationships. At the centre (m), BLTc and CLTc were moderately positively correlated (*r* = .453, *p* < .01), suggesting co-thickening of bone and cartilage. At r2, BLTc and CLTc were moderately negatively correlated overall (r 0 − 0.483, *p* < .01), driven primarily by FP2 (*r* = − .644, *p* < .01), consistent with a local trade-off between tissues. At r1, no consistent association emerged. For areas, BAc and CAc did not correlate, indicating that bone and cartilage areas varied largely independently, even though total area necessarily tracked both components.

## Discussion

### Histological composition of the stapes footplate

Previous studies on stapes morphology have concentrated mainly on external structure, employing light microscopy^13,14^ or micro-computed tomography (micro-CT) with a resolution limit of 6 μm^15–17^. Ultra-high-resolution CT imaging has also been reported by Tang et al.^18^. Emerging X-ray scattering–based approaches (e.g., SAXS tensor tomography) may further extend 3D microstructural characterization beyond morphology by providing orientation-sensitive information in mineralized tissues^19^. However, these methods do not allow for a detailed histological evaluation of the footplate. Until now, no comprehensive data have been available concerning the precise tissue distribution within the human stapes footplate, despite its critical relevance to reconstructive middle ear surgery and, ultimately, to hearing restoration.

This study presents the first detailed qualitative and quantitative analysis of the histological architecture of the stapes footplate. The findings contribute fundamentally to our understanding of its functional mechanics and have direct implications for the surgical placement and long-term success of TORP.

### Tissue layering and distribution—longitudinal sections

The footplate shows a bilayered histological structure in the majority (99.3%) of cases, composed of a cartilage layer facing the vestibule and a bone layer directed towards the tympanic cavity. These layers are covered by a unicellular submucosal layer of middle ear mucosa. In agreement with previous observations^20,21^, the present data show considerable heterogeneity in tissue structure in individual cases. Rarely identified patterns include bone completely surrounded by cartilage, a layer of bone sandwiched between layers of cartilage, and areas where there is no bone component at all (Fig. 4). However, the present results show that these variants are the exception to the histological footplate structure.

Tissue thickness varied significantly across short distances, even at identical anatomical landmarks (m, s1, s2, t1, t2) in successive sections. However, TLTl remained relatively consistent, with BLTl and CLTl demonstrating an inverse relationship.

At the central point (m), TLTl averaged 118.06 μm ± 46.35, with 61% cartilage and 39% bone. Towards the stapes crura, both layers thickened. At s1 and s2, TLTl was 65% and 42% larger than centrally, and BLTl was disproportionately larger (158% and 74%, respectively), indicating regional adaptation to higher mechanical load in the intact stapes. In contrast, CLTl showed similar values or only marginally larger values. This centre–edge difference in thickness is in line with imaging work that reports non-uniform footplate thickness with regional maxima near the crura^22^. Thus, the peripheral increase in TLTl was primarily bone-driven, whereas cartilage proportions were smaller, from 59% centrally to as low as 37%. This pattern is consistent with higher peripheral mechanical demands imposed by the stapedial annular ligament and the stapes’ mixed piston/rocking motions, which can concentrate stresses at the footplate edge^23–25^.

Tissue area measurements confirmed this trend. Bone occupied 39% of the total area centrally, increasing to 64% and 51% at s1 and s2. Cartilage area remained nearly unchanged, reinforcing the observation that bone dominates in load-bearing regions.

Statistically, position effects were supported by repeated-measures ANOVA: TLTl and BLTl at the center (m) differed from the edge s1 (*p* < .001 for both), whereas CLTl showed no positional effect. Treating s1 and s2 as unlabeled edges (orientation not preserved), paired TOST with a pre-specified margin (Δ = 50 μm) indicated equivalence between edges for TLTl and CLTl, but not for BLTl (mean s1–s2 = + 37.6 μm; 90% CI − 11.4 to 86.5). Together, these findings indicate a centre–edge gradient predominantly driven by bone, while cartilage thickness remains comparatively uniform; at the same time, bone exhibits between-footplate variability large enough to preclude claiming edge equivalence at this margin.

Extending the correlation analysis to planar areas, total area (TA) was tightly coupled to bone area (BA) across positions and subgroups, whereas the cartilage area (CA) contribution was more position- and subgroup-dependent. BA and CA were frequently negatively correlated, consistent with local trade-offs between bone and cartilage contributions to the same footprint. Mirrored-position symmetry was stronger for BA and TA (s1–s2 and t1–t2) but weaker for CA, indicating that side-to-side symmetry of planform is largely bone-driven, with cartilage adding local variability.

Taken together with the thickness results, these area data support a two-component allocation model: bone sets the baseline geometry (driving TA and TLTl, particularly at the edge), while cartilage acts as a local modulator that either supplements (e.g., FP6: positive CLTl–TLTl and strong CA–TA) or substitutes for bone (e.g., FP1/FP3: negative BLTl–CLTl and BA–CA). This compensatory role of cartilage provides a parsimonious explanation for the observed coexistence of pattern-level symmetry with non-equivalence in BLTl: redistribution of cartilage can stabilize TLTl across sides despite side-to-side differences in bone thickness. Centrally, where BLTl and CLTl co-thickened and both loaded TLTl, the positive CA–TA alongside strong BA–TA suggests coordinated expansion of both tissues to maintain effective cross-section and stiffness.

Functionally, a bone-dominated planform with cartilage fine-tuning at the edge is compatible with load sharing and sealing at the annular region^24,26^, while subgroup-dependent cartilage contributions (e.g., FP6) likely reflect micro-mechanical demands rather than active adult remodelling—consistent with the limited post-childhood remodelling of the ossicles^25,27^.

### Tissue distribution—cross sections

Cross-sectional analysis indicated a more homogeneous tissue distribution than in longitudinal planes. Centrally, TLTc averaged 73.44 μm ± 12.10, with cartilage contributing 58% and bone 42%. At the edge (r1, r2), TLTc differed only slightly (7% and 2.4%), whereas BLTc was smaller at r2 (= 11%) than at r1 (= 2.5%), with cartilage exhibiting only small regional variation.

Extending the correlation analysis, mirrored edges were symmetric for totals driven by cartilage: TLTc and CLTc correlated across r1–r2 (*p* = .040), and the area analogues TAc and CAc likewise showed symmetry (*p* = .034), despite the absence of superior/inferior labelling. In contrast, BLTc did not express r1–r2 symmetry, pointing to greater bone variability at the edge.

Centrally (m), BLTc and CLTc were moderately positively correlated (*r* = .453, *p* < .01), consistent with co-thickening of bone and cartilage. At r2, BLTc and CLTc were moderately negatively correlated overall (*r* = − .483, *p* < .01), driven by FP2 (*r* = − .644, *p* < .01), indicating a local trade-off between tissues under edge-specific constraints; no consistent association emerged at r1. For areas, BAc and CAc did not correlate, implying that bone and cartilage planform areas vary largely independently, even though TAc necessarily tracks both components.

Together, these cross-sectional patterns complement the longitudinal findings: bone largely sets the baseline geometry, but shows greater regional/asymmetric variability, while cartilage acts as a symmetry-preserving modulator at the edge (r1–r2 symmetry in CLTc/CAc and totals), and co-expands centrally with bone. The r2 trade-off (notably in FP2) suggests position-specific mechanical demands or spatial constraints that favor substitution (bone vs. cartilage) rather than uniform co-growth. Functionally, a bone-defined cross-section with cartilage fine-tuning reconciles the coexistence of pattern-level symmetry with non-equivalence in BLT and is compatible with the limited post-childhood remodelling of the ossicles^21^.

### Surgical implications: prosthesis placement and mechanical considerations

In TORP procedures, the mechanical function of the ossicular chain is bypassed. Force transmission is thus directly channelled from the prosthesis through the footplate to the inner ear. For optimal sound transmission, the prosthesis should ideally contact the central footplate region directly on the bone surface, without interposed soft tissue, to avoid acoustic attenuation^10,28^.

However, this area is structurally the most delicate. The minimal BLT measured was 2.93 μm (mean ± SD: 8.16 μm ± 2.64) in longitudinal and 6.21 μm (mean ± SD: 12.04 μm ± 8.24) in cross sections. The thinnest total layer thickness was 30.03 μm and 24.92 μm, respectively. Published work indicates that human auditory ossicles exhibit marked osteocyte depletion and lack significant bone remodelling capacity after early development^27,29^. This suggests that the extremely thin central bone layer of the footplate may have limited potential for osseointegration or regeneration, an important consideration when planning TORP anchoring.

Current guidelines recommend a prosthetic footplate diameter of 400–600 μm for effective sound transfer^30–32^. Yet, our data show that within this attachment area, the bone and cartilage layers are much thinner—posing a risk of fracture and subsequent perilymphatic fistula with inner ear exposure.

To mitigate these risks, alternative prosthesis designs have been developed. The Omega-Connector^®^ employs a titanium anchor placed on the footplate, with the TORP mounted in a secondary procedure^33^. Another approach involves a footplate anchor with a biomodal design, surface-modified to increase contact area and enhance osseointegration using bioactive coatings^7^. These strategies aim to distribute mechanical forces more evenly and improve implant stability and long-term auditory outcomes.

### External footplate morphology and clinical relevance

The external dimensions of the stapes footplate in this study align with existing literature (see Table 1. The intercrural length ranged from 2,532.23 μm to 2,721.23 μm (mean: 2.63 mm ± 0.13), while footplate width varied from 996.76 μm to 1,125.27 μm (mean: 1.06 mm ± 0.09). Variability is likely due to methodological differences – explantation and histological processing in this study, versus imaging-based measurements in others^17^.

**Table 1:** Summary of studies measuring stapes footplate length: mean ± SD, range (mm), and measurement methods used.

References	Numbers of stapes	Mean value of the total length (in mm)	Range of the total length (in mm)	Measuring method
^Anson (1939)[38]^	*n* = 18	2.47	2.0–2.71	Manual measurement in reflected light microscopy
^Dass et al. (1966) [12]^	*n* = 162	2.79	2.1–3.1	Manual measurement in reflected light microscopy
^Wadhwa et. al. (2005)[39]^	*n* = 10	2.97 ± 0.31	2.64–3.29	Software-supported reflected light microscopy
^Faragani & Nooradipur 2008[40]^	*n* = 12	2.29 ± 0.43	1.93–3.05	Electron microscopy
^Sim et al. (2013)[17]^	*n* = 40	2.81 ± 0.16	2.75–2.82	Micro-CT
^Calligas et al. (2008)[41]^	*n* = 10	2.71	2.52–2.97	Micro-CT
^Gong et al. (2023)[15]^	*n* = 30	2.93 ± 0.17	2.87–3.00	Micro-CT
Own study	*n* = 7	2.63 ± 0.13	2.53–2.72	Software-supported reflected light microscopy (histological)

Surface irregularities were confirmed by scanning electron microscopy, revealing convex central areas and flatter, concave regions near the annular ligament^12,34,35^. These findings corroborate previous reports^12,17,35^ and reflect observed variations in histological layer thickness. Such morphological diversity complicates preoperative planning, often requiring intraoperative adaptation to ensure prosthesis stability and compatibility^36^.

In some cases, anatomical constraints may necessitate prosthesis modification or the use of alternative coupling mechanisms such as the Omega-Connector^®^. Given spatial limitations in the middle ear cavity, a degree of individualised prosthesis design is essential.

The data also show that all previous experimental studies on footplate fractures with total prostheses are only of very limited significance^34,37^. Until now, fracture tests have never been correlated with the bone and cartilage thickness at the fracture line in the specific individual case - a necessity that arises inevitably from the data presented here.

### Functional and biomechanical implications of the bilaminar footplate structure

The histological evidence of a bilaminar structure, comprising bone and cartilage, within the stapes footplate elucidates its anatomical complexity and suggests potential biomechanical implications in the intact chain and after reconstruction. The medial cartilage layer, although not interposed at the prosthesis–bone contact (Fig. 2), could still influence the footplate’s overall bending stiffness and energy dissipation as part of a bilayer composite. Accordingly, we do not claim a direct buffering effect at the TORP–bone interface. Any indirect redistribution of loads attributable to the cartilage layer remains hypothesis-generating and would require biomechanical modelling (e.g., laminate/composite-plate analyses) to be substantiated. This interpretation aligns with our main conclusion that the peripheral planform is bone-dominated, with cartilage acting as a local modulator rather than implying active adult remodelling.

A related anatomical aspect, whether fibres of the stapedial annular ligament insert into cartilage or bone, was not systematically assessed in our series; we therefore refrain from drawing conclusions about insertion patterns and identify this as a question for targeted future work.

Together, these results offer a coherent rationale for the coexistence of bone and cartilage in the footplate and point to a biomechanical arrangement well-suited for sound conduction, mechanical stability, and injury mitigation. They also motivate further work on the dynamic interactions between the footplate and the annular ligament, particularly in pathological or surgically altered states, to inform prosthesis design, optimise implant placement, and improve long-term outcomes. As related anatomical detail, the specific insertion pattern of annular-ligament fibres (cartilaginous vs. bony) was not systematically assessed here; we therefore refrain from inferences on this feature and identify it as a priority for future targeted studies.

### Study limitations

This study is limited by the relatively small sample size and by the loss of anterior–posterior orientation during explantation and processing. These constraints affect generalisability and preclude directional comparisons between the anterior and posterior footplate edges, a distinction that is likely clinically relevant for TORP placement. Accordingly, we refrain from anterior–posterior specific recommendations.

Despite these limitations, the work provides value by delivering co-registered quantitative measurements of bone (BLT and BA) and cartilage (CLT and CA) across central and peripheral positions, by demonstrating a bone-dominated peripheral gradient with comparatively uniform cartilage thickness, and by supplying correlation matrices and equivalence tests that help separate centre–edge effects from between-footplate variability. These results offer a transparent baseline for hypothesis generation and for planning orientation-preserving studies.

Looking ahead, the most impactful steps are to preserve anatomical orientation throughout, for example by using simple anterior and posterior fiducials and consistent photographic documentation, to increase sample size through multi-centre collaboration with prespecified power and equivalence margins informed by the present effect sizes, and to link histology to three-dimensional context via high-resolution imaging prior to sectioning with subsequent registration. In this context, orientation-sensitive 3D X-ray techniques such as SAXS tensor tomography have been proposed as a future tool for structural analysis in life-science specimens and could be explored for ossicular tissues^19^. In analysis, hierarchical or mixed-effects models can better separate positional from between-specimen variance, and focused reproducibility checks, including observer agreement and sensitivity analyses for layer segmentation, will strengthen inference. Finally, coupling the measured layer metrics to mechanical models, for example laminate or finite-element analyses, can test how potential anterior–posterior differences, once orientation is preserved, would influence load sharing and optimal TORP seating.

In sum, although orientation loss and sample size limit anterior–posterior inference and generalisability, the present data establish quantitative patterns, namely bone-driven peripheral thickening with relatively uniform cartilage, that are clinically informative as a starting point. Orientation-preserving, adequately powered, and model-integrated studies are needed to resolve anterior–posterior differences with direct relevance for TORP placement.

## Conclusion

This study provides the first systematic, spatially resolved histological characterisation of the human stapes footplate. A largely consistent bilaminar architecture was identified, with cartilage facing the vestibule and bone toward the tympanic cavity. Quantitatively, bone predominated toward the periphery near the stapes crura, whereas cartilage thickness was comparatively uniform and contributed to central co-thickening. Correlation patterns and centre–edge comparisons helped disentangle positional effects from between-specimen variability, yielding a robust description of regional tissue allocation.

These findings have direct implications for reconstructive middle ear surgery. In total ossicular replacement, load transmission proceeds from the prosthesis through the footplate to the inner ear, which makes local thickness and composition critical for safe and effective coupling. The central region is acoustically favourable yet structurally delicate, as indicated by very thin bone and total thickness within typical prosthetic footprints. Our data therefore support prosthesis strategies and placements that account for local geometry and material distribution, and they provide quantitative parameters for biomechanical modelling and device optimisation.

The work also offers a framework for future studies that link histology with function. Orientation-preserving sampling, larger cohorts, and three-dimensional imaging with registration can refine anterior–posterior comparisons that are clinically relevant for prosthesis seating. Hierarchical statistical approaches and targeted mechanical models, for example laminate or finite-element analyses parameterised by measured layer metrics, can test how regional tissue allocation influences load sharing, stability, and long-term outcomes. Although sample size and loss of anterior–posterior orientation limit generalisability and directional inference, the present data establish clinically meaningful patterns and a transparent baseline for subsequent, model-integrated research aimed at improving prosthesis design, placement, and durability.

## Supplementary Information

Below is the link to the electronic supplementary material.

Supplementary Material 1

Supplementary Material 2

## Data Availability

All microscopy images and raw measurement data supporting the results of this manuscript are stored on secure internal servers. Due to institutional data protection policies and ethical considerations regarding human tissue specimens, these data are not publicly available. Access can be granted upon reasonable request to the corresponding author.

## Abbreviations

FP: Stapes footplate
SC: Stapes crura
TORP: Total ossicular replacement prosthesis
BLTl: Bone layer thickness in longitudinal section
BLTc: Bone layer thickness in cross section
CLTl: Cartilage layer thickness in longitudinal section
CLTc: Cartilage layer thickness in cross section
TLTl: Total tissue layer thickness in longitudinal section
TLTc: Total tissue layer thickness in cross section
CAl: Cartilage area in longitudinal section
CAc: Cartilage area in cross section
BAl: Bone area in longitudinal section
BAc: Bone area in cross section
TAl: Total tissue area in longitudinal section
TAc: Total tissue area in cross section
MV: Mean value
SD: Standard deviation
