# Supplementary material for: The quantitative and qualitative histomorphological structure of human stapes footplate
Source: Sci Rep. 2026 Mar 19;16:9537. doi: 10.1038/s41598-026-43700-8 (PMC13004908; doi:10.1038/s41598-026-43700-8)
Supplement: Supplementary file 1 — Supplementary Material 1 [file 41598_2026_43700_MOESM1_ESM.docx]

Supplement Material


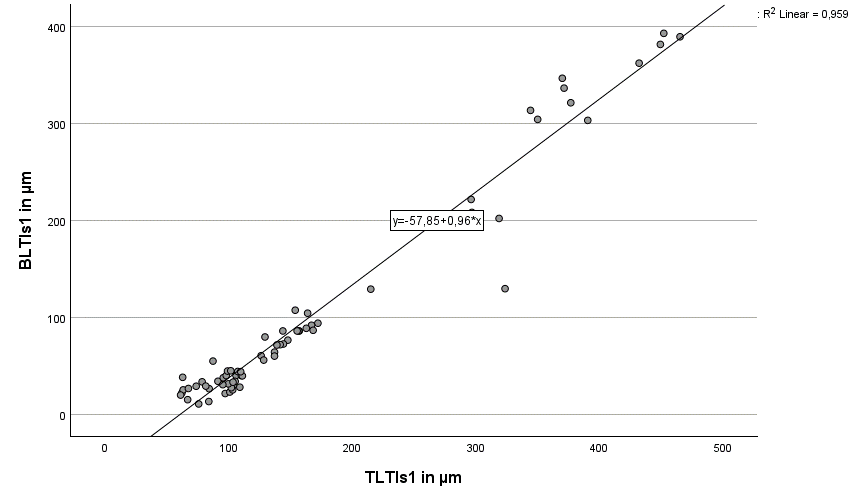


Supplementary Figure S1. Longitudinal correlation at measurement point s1 (representative): bone layer thickness (BLTl) vs. total layer thickness (TLTl). Linear fit: y = −57.85 + 0.96x, R² = 0.959; p < 0.001. Points represent individual measurements; the line shows the ordinary least squares (OLS) regression.


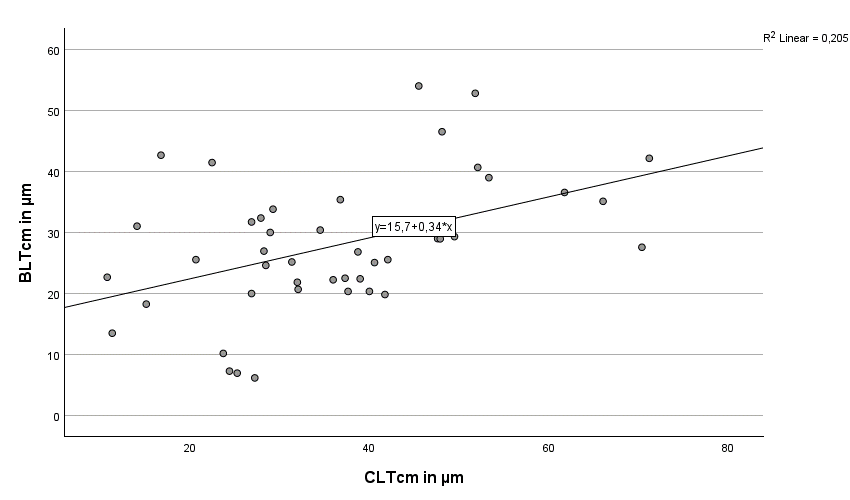


Supplementary Figure S2. Cross-sectional correlation at measurement point m: bone layer thickness (BLTc vs. cartilage layer thickness (CLTc​). Linear fit: y = 15.7 + 0.34x, R² = 0.205; p < 0.01. Points represent individual measurements; the line shows the ordinary least squares (OLS) regression.


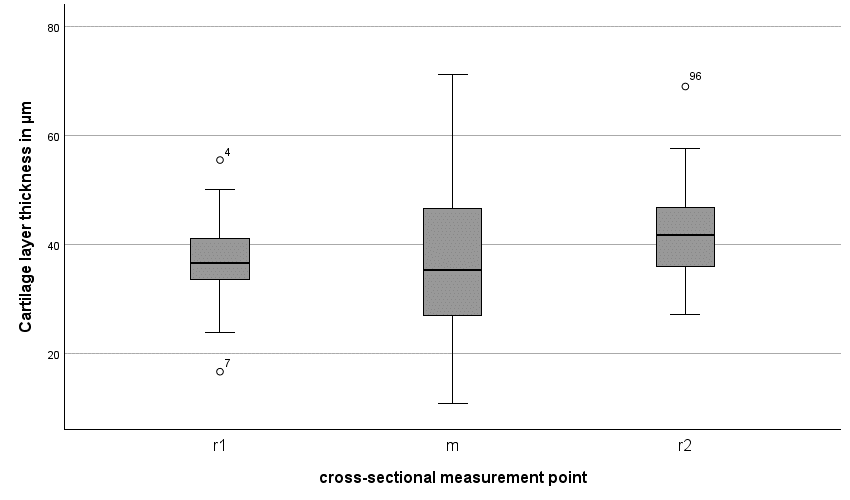


Supplementary Figure S3. Boxplots of cartilage layer thickness (CLTc) in cross sections by measurement point (r1, m, r2). Distributions summarize all section-level measurements (n = 44 per point; footplates: FP1 n = 12, FP2 n = 32). Boxes show the median and interquartile range (IQR); whiskers extend to 1.5×IQR; circles denote outliers.

Supplementary Table S1. Longitudinal analysis (s1, t1, m, t2, s2): layer thicknesses and areas with correlation analyses. Means ± SD for longitudinal total layer thickness (TLTl), bone (BLTl), cartilage (CLTl), and the corresponding areas (TAl, BAl, CAl); additionally stratified by footplates (FP1–FP6). Reported are Pearson correlations between parameters with r values and significance.


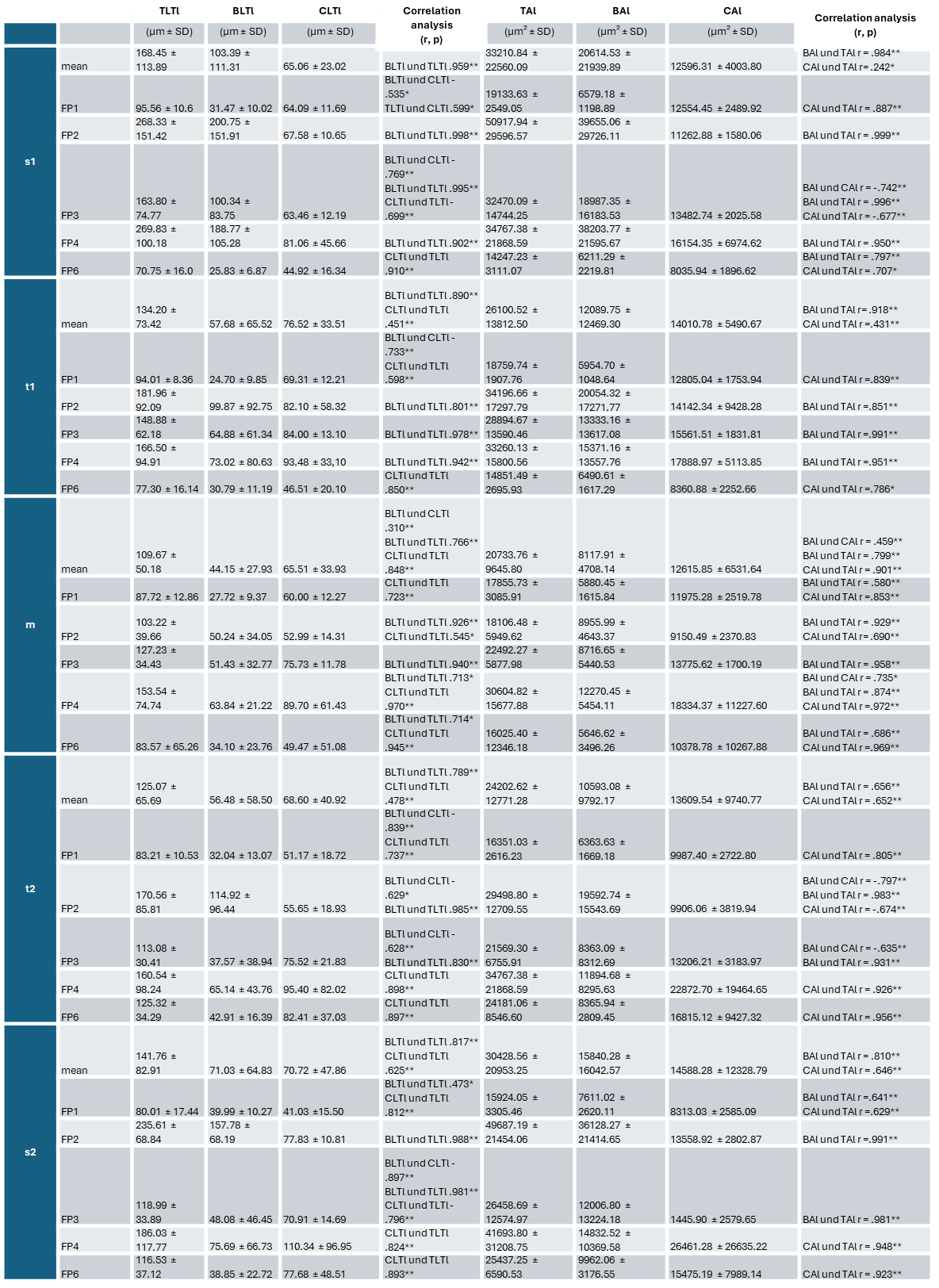


Footnotes: r = Pearson’s r; p < 0.05 (*), p < 0.01 (**); n.s. = not significant. Units: thickness in µm, areas in µm².

Supplementary Table S2. Cross-sectional analysis (r1, m, r2): layer thicknesses and areas with correlation analyses. Means ± SD for total layer thickness (TLTc), bone layer thickness (BLTc), cartilage layer thickness (CLTc), and the corresponding areas (TAc, BAc, CAc) at measurement points r1, m, and r2; additionally stratified by footplates (FP1/FP2). Reported are Pearson correlations between parameters with r values and significance.

| Measurement Point | | TLTc | BLTc | CLTc | Correlation analysis  (r, p) | TAc | BAc | CAc | Correlation analysis  (r, p) |
| --- | --- | --- | --- | --- | --- | --- | --- | --- | --- |
|  |  | (µm ± SD) | (µm ± SD) | (µm ± SD) |  | (µm² ± SD) | (µm² ± SD) | (µm² ± SD) |  |
| r1 | mean | 65.60 ± 13.93 | 28,46 ± 13,29 | 37,14 ± 7,33 | TLT and BLT r=.856** TLT and CLT r=.349* | 13764.79 ± 4491.23 | 6195.15 ± 2065.29 | 7569.64 ± 1426.57 | TLT and BLT r=.690** TLT and CLT r=.506* |
|  | FP1 | 73,09 ± 14,26 | 33,9 ± 10,98 | 39,19 ± 11,6 | n.s. | 15085.66 ± 2558.83 | 6934.30 ± 3041.63 | 8151.35 ± 2637.25 | n.s. |
|  | FP2 | 63,5 ± 13,31 | 26,93 ± 13,63 | 36,57 ± 5,75 | TLT and BLT r=.909** | 12443.92 ± 1336.78 | 5455.10 ± 1088.95 | 6987.92 ± 874.89 | TLT and BLT r=.758** TLT and CLT r=.584* |
| m | mean | 64.12 ± 22.19 | 27,87 ± 11,04 | 36,24 ± 14,89 | TLT and BLT r=.801** TLT and CLT r=.896** BLT and CLT r=.453** | 16292.11 ± 6379.46 | 6874.95 ± 2427.17 | 9417.16 ± 5850.32 | TLT and BLT r=.394** TLT and CLT r=.977* |
|  | FP1 | 93,94 ± 11,41 | 38,45 ± 9,17 | 55,46 ± 9,33 | TLT and BLT r=.608* TLT and CLT r=.625* | 19436.73 ± 1855.91 | 8591.22 ± 1633.17 | 10845.51 ± 1106.03 | TLT and BLT r=.806** |
|  | FP2 | 52,93 ± 12,78 | 23,89 ± 8,91 | 29,04 ± 8,95 | TLT and BLT r=.714* TLT and CLT r=.717* | 13147.47 ± 10903.37 | 5158.69 ± 1385.10 | 7988.80 ± 10594.61 | TLT and CLT r=.992** |
| r2 | mean | 68.91 ± 11.40 | 26.78 ± 12.84 | 42.14 ± 8.10 | TLT and BLT r=.783** BLT and CLT r=-.483** | 14909.56 ± 3921.47 | 6023.55 ± 1945.63 | 8250.84 ± 1161.73 | TLT and BLT r=.782** TLT and CLT r=.433* |
|  | FP1 | 75,79 ± 13,48 | 29,73 ± 11,35 | 46,06 ± 4,98 | TLT and BLT r=.934** | 15053.82 ± 2683.23 | 6142.86 ± 2095.61 | 8910.96 ± 1723.53 | TLT and BLT r=.767** |
|  | FP2 | 66,98 ± 10,16 | 25,94 ± 13,28 | 41,04 ± 8,52 | TLT and BLT r=.767** BLT and CLT r=-.644** | 13494.95 ± 1644.50 | 5904.24 ± 1795.65 | 7590.71 ± 965.04 | TLT and BLT r=.846** |

Footnotes: r = Pearson’s r; p < 0.05 (*), p < 0.01 (**); n.s. = not significant. Units: thickness in µm, areas in µm².

Supplementary Table S3. Longitudinal between-site correlations (Pearson’s r).

|  |  | **s1 vs. s2** | **t1 vs. t2** |
| --- | --- | --- | --- |
| Thickness | BLTl | .544** | .508** |
|  | CLTl | n.s. | n.s. |
|  | TLTl | .651** | .564** |
| Area | BAl | .651** | .574** |
|  | CAl | .435** | n.s. |
|  | TAl | .682** | .555** |

Footnotes: r = Pearson’s correlation coefficient. ** = p < 0.01. n.s. = not significant.

Supplementary Table S4. Cross-sectional between-site correlations (Pearson’s r).

|  |  | **r1 vs. r2** |
| --- | --- | --- |
| Thickness | BLTc | .353* |
|  | CLTc | n.s. |
|  | TLTc | .378* |
| Area | BAc | .409** |
|  | CAc | .628** |
|  | TAc | .667** |

Footnotes: r = Pearson’s correlation coefficient. ** = p < 0.01. n.s. = not significant.
